# Gut microbiota of newborn piglets with intrauterine growth restriction have lower diversity and different taxonomic abundances

**DOI:** 10.1111/jam.14304

**Published:** 2019-06-07

**Authors:** W. Zhang, C. Ma, P. Xie, Q. Zhu, X. Wang, Y. Yin, X. Kong

**Affiliations:** ^1^ Hunan Provincial Key Laboratory of Animal Nutritional Physiology and Metabolic Process, Key Laboratory of Agro‐ecological Processes in Subtropical Region, National Engineering Laboratory for Pollution Control and Waste Utilization in Livestock and Poultry Production Institute of Subtropical Agriculture, Chinese Academy of Sciences Changsha Hunan China; ^2^ University of Chinese Academy of Sciences Beijing China; ^3^ Research Center of Mini‐Pig Huanjiang Observation and Research Station for Karst Ecosystems, Chinese Academy of Sciences Huanjiang China

**Keywords:** diversity, gut microbiota, intrauterine growth restriction, metabolism, piglets, small intestine

## Abstract

**Aim:**

Intrauterine growth retardation (IUGR) is a prevalent problem in mammals. The present study was conducted to unveil the alterations in intestinal microbiota in IUGR piglets.

**Methods and Results:**

We identified the alterations of small intestinal microbiota in IUGR piglets on 7, 21 and 28 days of age using 16S rRNA sequencing. The results showed that IUGR piglets had a decreased alpha diversity of jejunum microbiota at 7 and 21 days of age; had lower abundances of *Bacteroidetes* and *Bacteroides* in the jejunum at 7, 21 and 28 days of age, *Oscillibacter* in the jejunum at 21 days of age, and *Firmicutes* in the ileum at 21 days of age; whereas they had higher abundances of *Proteobacteria* and *Pasteurella* in the ileum at 21 days of age and *Escherichia–Shigella* in the jejunum at 28 days of age. Correlation analysis showed that *Bacteroides, Oscillibacter* and *Ruminococcaceae_UCG‐002* compositions were positively associated with the body weight (BW) of IUGR piglets, nevertheless *Proteobacteria* and *Escherichia–Shigella* relative abundances were negatively correlated with the BW of IUGR piglets. Gene function prediction analysis indicated that microbiota‐associated carbohydrate metabolism, lipid metabolism, glycan biosynthesis and metabolism, amino acid metabolism, and xenobiotics biodegradation and metabolism were downregulated in the IUGR piglets compared to control piglets.

**Conclusions:**

The present study profiled the intestinal microbiota of newborn piglets with IUGR and the newborn IUGR piglets have lower diversity and different taxonomic abundances. Alterations in the abundances of *Bacteroidetes*, *Bacteroides*, *Proteobacteria Escherichia–Shigella* and *Pasteurella* may be involved in nutrient digestion and absorption, as well as the potential mechanisms connecting to the growth and development of IUGR in mammals.

**Significance and Impact of the Study:**

The small intestinal microbiota were highly shaped in the IUGR piglets, which might further mediate the growth and development of IUGR piglets; and the gut microbiota could serve as a potential target for IUGR treatment.

## Introduction

The reproductive performance of sows directly determines the production efficiency and economic benefits in the modern swine industry. Although the reproductive performance can be improved by dietary nutrients, feeding strategies and genetic breeding, low‐birth weight widely occurs in newborn animals (Milligan *et al.* 2002; Quiniou *et al.* 2002; Campos *et al.* 2012; Matheson *et al.* 2018). Intrauterine growth restriction (IUGR) has been defined as the impaired growth and development of the mammalian embryo/fetus or its organs during pregnancy, which seriously affects animal production and health (D'Inca *et al.* 2010). For example, IUGR animals generally have a reduced neonatal survival rate, gut dysfunction, a low efficiency of nutrient utilization and postnatal long‐term growth limitation (Wu *et al.* 2006). Previous studies confirmed that the impaired development and functions of the intestine in IUGR piglets result from changes in the developmental pattern of the intestinal structure, and transcriptomic and proteomic profiles (Wang *et al.* 2008; D'Inca *et al.* 2011; Dong *et al.* 2016).

The mammalian gastrointestinal tract harbours a complex and diverse microbial community, which plays crucial roles in host digestion, metabolism, immune function and redox balance (Turnbaugh *et al.* 2009; Hooper *et al.* 2012; Karlsson *et al.* 2013; Zhang *et al.* 2013; Yin *et al.* 2018). Early life colonization and development of the gut microbiota in neonates highly shapes the host’s metabolism and health (Ottman *et al.* 2012; Matamoros *et al.* 2013). This process can be influenced by various factors, such as the ways of delivery, diet composition during infancy, antibiotic usage and the host’s health status (Vaishampayan *et al.* 2010; Nicholson *et al.* 2012; Liu *et al.* 2018b). The microbial composition varies along the different regions of the gastrointestinal tract. For example, the composition of the microbiome is markedly differentiated among the small intestine, large intestine, and faeces, whereas the microbial profile of the large intestine is more similar to that of faeces (Zhao *et al.* 2015). In addition, the microbial community in the small intestine has a lower diversity and abundance than the colonic microbiota (Donaldson *et al.* 2016). Li *et al. *(2018) showed that low birth weight piglets have a different faecal microbial community structure and metabolite profiles, suggesting the gut microbial potentially associated with the impaired growth and development of these piglets. However, the composition of the small intestinal microbiota in IUGR piglets still remains unknown.

The small intestine is the major organ involved in digestion, absorption, metabolism and immune function. The microbiota in small intestine are critical transducers of dietary signals that allow the host to adapt to variations in lipid digestion and absorption (Martinez‐Guryn *et al.* 2018). The jejunum has a unique acute role in the gut response to luminal microbe—diet interplay, and microbiota in the jejunum strongly affects glucose and energy metabolism (El Aidy *et al.* 2013). Therefore, the small intestine microbiota also have a profound impact on host. He *et al. *(2011) confirmed that IUGR piglets have a distinctive metabolism in jejunum which contributes to impaired growth and jejunal function. But this study did not investigate the microbial profiles. Thus, we hypothesize that there are differences in microbial community in the small intestine between IUGR piglets and normal birth weight (NBW) piglets, which is associated with the impacted growth performance. The present study was conducted to characterize the microbiota profiles of the small intestine between IUGR piglets and control subjects in order to identify intestinal bacterial makers associated with IUGR.

## Materials and methods

### Ethical approval

This study was carried out in accordance with the Chinese guidelines for animal welfare and experimental protocols and was approved by the Animal Care and Use Committee of Institute of Subtropical Agriculture, Chinese Academy of Sciences, No. ISA‐2017‐016.

### Experimental design and sampling

In the present study, Large White × Landrace piglets were obtained from sows of an experimental herd located in Yong’an Town, Liuyang City, Hunan Province, China. Piglets with a birth weight higher than the mean birth weight were classified as NBW piglets, and piglets within the 10% lower mean birth weight were classified as IUGR piglets (Bauer *et al.* 1998). A total of 48 piglets were obtained from 24 litters, in which each contained one NBW piglet and one IUGR piglet. All suckling piglets were kept in a warm thermal container and fed by sows freely. The commercial creep feed was supplied from 5 days after birth. The piglets were weaned at 21 days of age and transferred into the nursery pens. The pigs had 24 h access to commercial weaning diet and water. No antibiotics were used during the entire experimental period. At 7 (7 days), 21 (21 days) and 28 (28 days) days after birth, 16 piglets (eight pairs of one NBW piglet and one IUGR littermate) were weighed and killed for sample collection 2 h after the last suckling. Briefly, the piglets were killed by exsanguination after general anaesthesia (intravenous injection of 4% sodium pentobarbital solution, 40 mg kg^−1^ BW), and then, the luminal contents of the jejunum (10 cm below the flexura duodenojejunalis) and ileum (10 cm above the ileo‐caecal junction) were sampled. The luminal content samples were stored at −80°C for subsequent analysis of gut microbial composition.

### DNA extraction and pyrosequencing

Microbial DNA was extracted from jejunum and ileum content samples using a HiPure Stool DNA Kit (Magen, Guangzhou, China) following the manufacturer’s instructions. The final DNA concentration and purification were determined using a NanoDrop 2000 UV‐vis spectrophotometer (Thermo Fisher Scientific, Waltham, MA), and DNA quality was checked by 1% agarose gel electrophoresis. The V3–V4 hypervariable regions of the bacterial 16S rRNA gene were amplified with the primers 338F (5′‐ACTCCTACGGGAGGCAGCAG‐3′) and 806R (5′‐GGACTACHVGGGTWTCTAAT‐3′) using a thermocycler PCR system (GeneAmp 9700; Thermo Fisher Scientific), as described by Yin *et al. *(2017). The PCR reactions were conducted using the following programme: 3‐min denaturation at 95°C; 27 cycles of 30 s at 95°C, 30‐s annealing at 55°C, and 45‐s elongation at 72°C and a final extension at 72°C for 10 min. PCR reactions were performed in a 20 μl mixture containing 4 μl 5× FastPfu buffer, 2 μl 2·5 mmol l^−1^ dNTPs, 0·8 μl each primer (5 μmol l^−1^), 0·4 μl FastPfu Polymerase and 10 ng template DNA, in triplicate. The resultant PCR products were extracted from a 2% agarose gel and further purified using the AxyPrep DNA Gel Extraction Kit (Axygen Biosciences, Union City, CA) and quantified using QuantiFluor™‐ST (Promega, Madison, WI) according to the manufacturer’s protocol. Purified amplicons were pooled in equimolar amounts and paired‐end sequenced (2 × 300) on an Illumina MiSeq platform (Illumina, San Diego, CA) at Shanghai Majorbio Bio‐pharm Technology Co., Ltd (Shanghai, China).

### Bioinformatics and statistical analysis

Raw fastq files were demultiplexed, quality‐filtered using Trimmomatic (Bolger *et al.* 2014) and merged using flash (Magoc and Salzberg 2011) with the following criteria: (i) the reads were truncated at any site with an average quality score <20 over a 50 bp sliding window; (ii) primers were matched allowing for two nucleotides to mismatch, and reads containing ambiguous bases were removed; (iii) sequences with an overlap longer than 10 bp were merged according to their overlap sequence. Operational taxonomic units (OTUs) were clustered with a 97% similarity cut‐off using uparse (ver. 7.1) (Edgar 2013), and chimeric sequences were identified and removed using uchime (Edgar 2010). The taxonomy of each 16S rRNA gene sequence was analysed using RDP Classifier algorithm (http://rdp.cme.msu.edu/) against the Silva (SSU123) 16S rRNA database using confidence threshold of 70%. The alpha diversity analysis included analysis of the Shannon index, Simpson index, Chao1 richness estimator and abundance‐based coverage estimator (ACE) metric. A beta diversity analysis was performed to investigate the structural variation in the microbial communities across samples using principal coordinate analysis (PCoA) based on unweighted UniFrac distance. Partial least squares discriminant analysis (PLS‐DA) based on unweighted UniFrac distance was also introduced as a supervised model to reveal the microbiota variation among groups. Taxonomic composition was investigated at the phylum and genus levels. The functional profiles of the microbial communities were predicted using Phylogenetic Investigation of Communities by Reconstruction of Unobserved States (PICRUSt) (Langille *et al.* 2013). All of these analyses were performed on the free online platform of Majorbio I‐Sanger Cloud Platform (http://www.i-sanger.com).

Mann–Whitney *U* test was used to test for significant differences among all data. Differences between the IUGR and NBW groups were considered significant at *P* < 0·05. Data were presented as means ± SEM. spss 22.0, Excel 2010, R package ggplot2, and GraphPad Prism ver. 6.0 (San Diego, CA) were used for data analysis and graph preparation.

## Results

### Body weight and average daily gain

**Figure 1:**
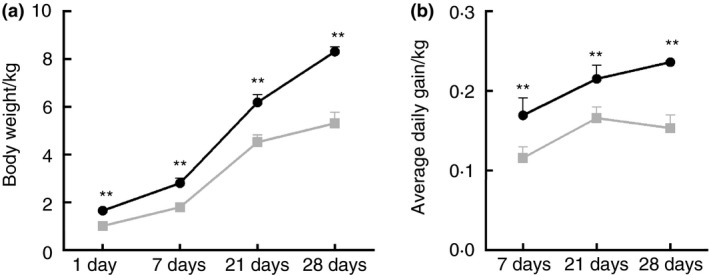
Differences in body weight (a) and average daily gain (b) between intrauterine growth retardation (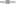] IUGR) piglets and normal birth weight ([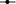] NBW) piglets. Asterisks indicate different from corresponding NBW group: *P < 0·05; **P < 0·01.

The birth weight of IUGR piglets was significantly lower (by 39%, *P* < 0·05) than that of NBW piglets (Fig. 1a). The BW of IUGR piglets at 7, 21 and 28 days of age was significantly lower (*P* < 0·05) by 36, 27 and 36%, respectively, than that of NBW piglets. In addition, the average daily gain was reduced (*P* < 0·05) by 32, 23 and 35%, respectively, compared to NBW piglets (Fig. 1b).

### Metadata and sequencing

**Figure 2:**
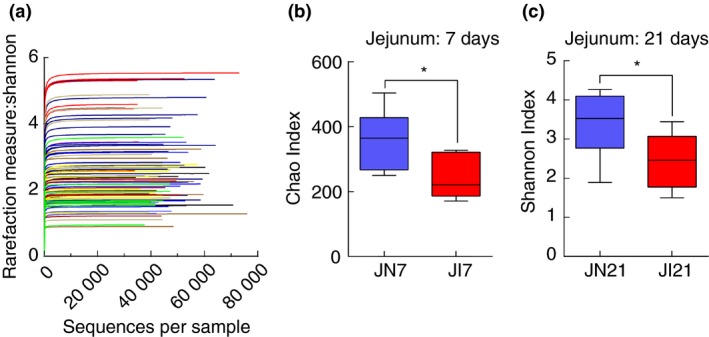
Differences in microbial community structures in small intestine between intrauterine growth retardation (IUGR) piglets and normal birth weight (NBW) piglets. (a): Rarefaction curve analysis was used to evaluate whether further sequencing would likely detect additional taxa, indicated by a plateau. JI7 (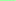), JN7 (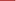), II7 (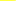), IN7 (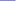), JI21 (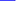), JN21 (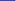), II21 (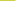), IN21 (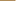), JI28 (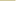), JN28 (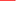), II28 (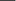), IN28 (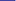) (b): Differences in bacterial diversity between the IUGR piglets and NBW piglets in jejunum luminal contents according to the Chao1 index on 7 days of age. (c) Differences in bacterial diversity between the IUGR piglets and NBW piglets in jejunum luminal contents according to the Shannon index on 21 days of age NBW (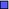), IUGR (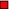). JI7 and JI21 represent samples obtained from jejunum luminal contents of IUGR piglets on 7 and 21 days of age, respectively. JN7 and JN21 represent samples obtained from jejunum luminal contents of NBW piglets on 7 and 21 days of age respectively. Asterisks indicate different from corresponding NBW group: *P < 0·05; **P < 0·01. [Colour figure can be viewed at http://wileyonlinelibrary.com]

In general, high‐throughput sequencing of 96 samples (including 48 jejunum content and 48 ileum content samples from piglets 7, 21 and 28 days of age) generated 4 842 943 high‐quality reads. A total of 1724 OTUs were obtained by clustering nonrepetitive sequences (excluding single sequences) based on 97% similarity, including 23 phyla, 42 classes, 79 orders, 140 families, 436 genera and 824 species. Each sample contained 47 047 OTUs and 50 447 sequences on average. The rarefaction curve (Fig. 2a) reached a plateau and the Good’s coverage for observed OTUs was 99·59 ± 0·02% (range of 99·26–99·88%, Table [Link]), which indicated a near‐complete sampling of the community.

### Microbial community diversity in the small intestine

Alpha diversity was measured using the Shannon, Simpson, ACE and Chao1 indexes (Fig. S1). There were no significant differences between Simpson indexes or ACE indexes of both the jejunum and ileum samples from the two groups. However, the IUGR group had a significantly lower (*P* < 0·05) Chao1 index for the jejunum than the NBW group at 7 days of age (Fig. 2b and Fig. S1), and the Shannon index of the jejunum was significantly lower than that of the NBW group at 21 days of age (Fig. 2c and Fig. S1). In addition, there was no significant difference in the diversity of the small intestine microbiota at 28 days of age between the two groups.

**Figure 3:**
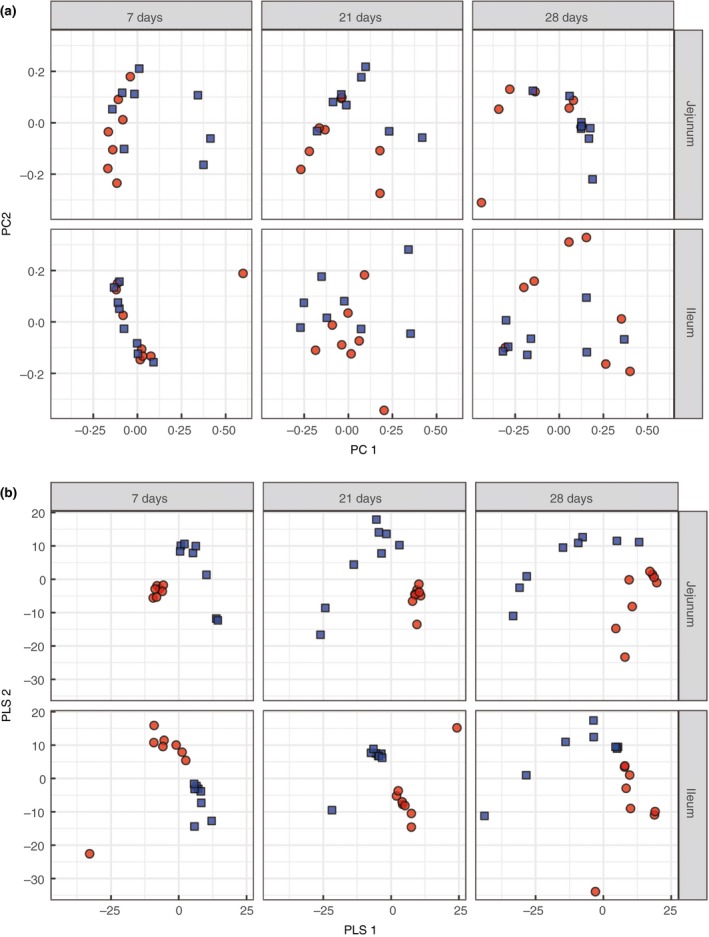
Differences in microbial community structures between intrauterine growth retardation (IUGR) piglets and normal birth weight (NBW) piglets. (a) Principal coordinate analysis (PCoA) on unweighted UniFrac distances between IUGR piglets and NBW piglets is shown along the first two principal coordinate (PC) axes. (b) Partial least square discriminant analysis (PLS‐DA) score plot of jejunum and ileum microbiota between the IUGR piglets and NBW piglets. Each symbol represents the gut microbiota of one piglet, red represents IUGR piglets and blue represents NBW piglets (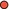 IUGR; 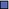 NBW). [Colour figure can be viewed at http://wileyonlinelibrary.com]

The dissimilarity of the bacterial community structure in the small intestine of the IUGR and NBW groups was measured by PCoA at OTU level. There was no obvious separation between the two groups (Fig. 3a). This was further investigated using PLS‐DA performed as a supervised analysis suitable for high‐dimensional data. The microbial community structure in both the jejunum and ileum was clearly separated and clustered into two groups at 7, 21 and 28 days of age, indicating significant differences in the small intestinal microbiota between the IUGR and NBW piglets (Fig. 3b).

### Microbial community composition in the small intestine

**Figure 4:**
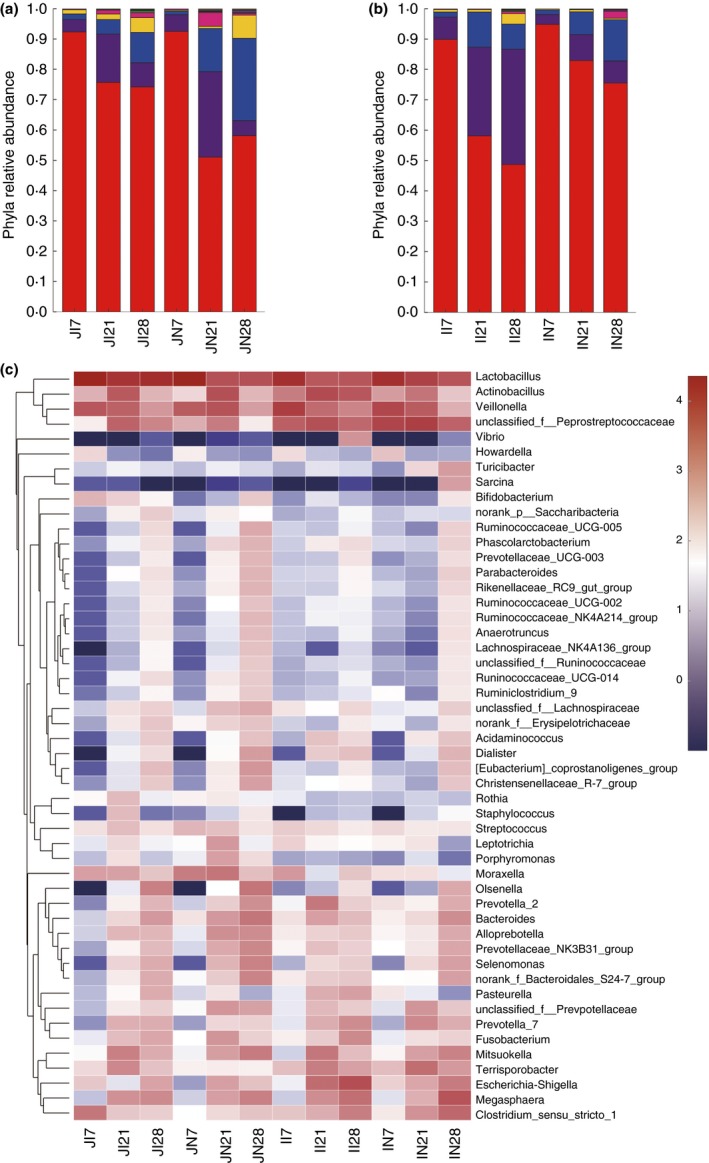
Microbial composition in small intestine of intrauterine growth retardation (IUGR) piglets and normal birth weight (NBW) piglets. The relative abundant of taxa > 0·01% at phylum are shown, and the top 50 abundant taxa at genus level are shown. (a): Microbial composition in jejunum of IUGR piglets and NBW piglets at phylum level (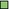 *Others;* 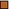 *Gracilibacteria;* 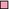 *SR1;* 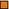 *Synergistetes;* 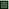 *Tenericutes;* 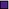 *Spirochaetae;* 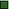 *Saccharibacteria;* 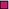 *Fusobacteria;* 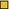 *Actinobacteria;* 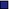 *Bacteroidetes;* 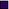 *Proteobacteria;* 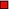 *Firmicutes*). (b): Microbial composition in ileum of IUGR piglets and NBW piglets at phylum level (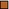 Others; 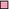 Synergistetes; 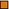 Tenericutes; 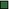 Saccharibacteria; 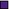 Spirochaetae; 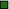 Cyanobacteria; 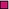 Actinobacteria; 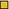 Fusobacteria; 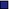 Bacteroidetes; 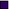 Proteobacteria; 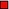 Firmicutes). (c): Microbial composition in jejunum and ileum of IUGR piglets and NBW piglets at genus level. JI7, JI21, JI28, II7, II21 and II28 represent samples obtained from jejunum and ileum luminal contents of IUGR piglets on 7, 21 and 28 days of age respectively. JN7, JN21, JN28, IN7, IN21 and IN28 represent samples obtained from jejunum and ileum luminal contents of NBW piglets on 7, 21 and 28 days of age respectively. [Colour figure can be viewed at http://wileyonlinelibrary.com]

The microbial communities of all the samples were analysed at the phylum and genus taxonomic levels (Fig. 4). *Firmicutes* was the dominant phylum in both the jejunum and ileum among the age groups. In the jejunum, *Firmicutes*, *Proteobacteria* and *Bacteroidetes* were the top three dominant phyla in the IUGR group at 7, 21 and 28 days of age, as well as in the NBW group at 7 and 21 days of age. The relative abundance of *Actinobacteria* increased and became one of the dominant phyla in the NBW group at 28 days of age. The relative abundance of *Bacteroidetes* and *Actinobacteria* increased continuously with the ages in the two groups. The relative abundance of *Proteobacteria* increased from 7 to 21 days of age, but then decreased at 28 days of age in the two groups. The relative abundance of *Firmicutes* decreased continuously with the ages in the two groups but increased at 28 days of age in the NBW group (Fig. 4a). Similarly, *Firmicutes*, *Proteobacteria* and *Bacteroidetes* were the top three abundant phyla in the ileum of the two groups. The abundance of *Firmicutes* decreased with age in the two groups. In addition, the relative abundance of *Proteobacteria* in the IUGR group and of *Bacteroidetes* in the NBW group both increased continuously with the age. The relative abundance of *Bacteroidetes* in the IUGR group and of *Proteobacteria* in the NBW group increased from 7 to 21 days of age but decreased at 28 days of age (Fig. 4b).

At the genus level, the bacterial distribution among samples was displayed as a heat map (Fig. 4c). In the jejunum, *Lactobacillus* was the most abundant genus in the IUGR and NBW groups at 7, 21 and 28 days of age, followed by *Veillonella* and *Clostridium_sensu_stricto_1* in the IUGR group, and *Veillonella* and *Moraxella* in the NBW group at 7 days of age; *Veillonella* and *Moraxella* in the IUGR and NBW groups at 21 days of age; and *Olsenella* and unclassified *Peptostreptococcaceae* in the IUGR group, and *Olsenella* and *Bacteroides* in the NBW group, at 28 days of age respectively. In the ileum, *Lactobacillus* was the dominant genus in the IUGR and NBW groups at 7 days of age, followed by *Veillonella* and unclassified *Peptostreptococcaceae*; at 21 days of age, *Actinobacillus*, unclassified *Peptostreptococcaceae* and *Lactobacillus* were the top three abundant genera in the IUGR group, and unclassified *Peptostreptococcaceae*, *Lactobacillus* and *Veillonella* were the dominant genera in the NBW group; at 28 days of age, *Escherichia–Shigella* was the most abundant genus in the IUGR group, followed by *Lactobacillus* and *Actinobacillus*, and *Megasphaera*, *Lactobacillus* and unclassified *Peptostreptococcaceae* were the top three abundant genera in the NBW group.

### Differences in microbial communities between IUGR and NBW piglets

**Figure 5:**
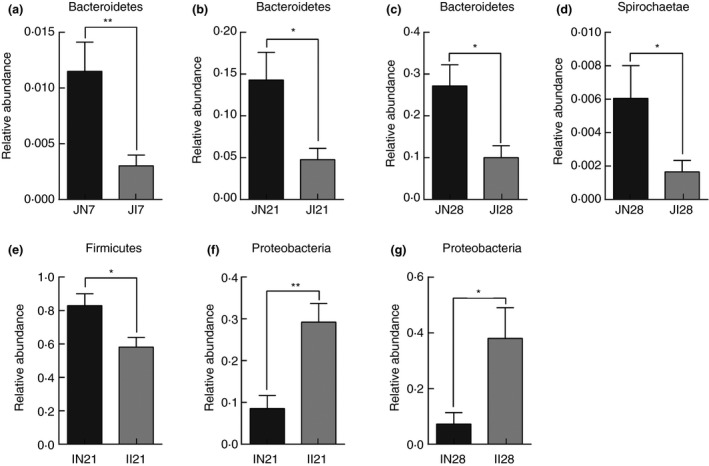
Differences in microbial abundance between intrauterine growth retardation (IUGR) piglets and normal birth weight (NBW) piglets at phylum level. (a–c): The relative abundance of *Becteroidetes* of jejunum on 7, 21 and 28 days of age respectively; (d): The relative abundance of *Spirochaetae* of jejunum on 28 days of age; (e): The relative abundance of *Firmicutes* of ileum on 21 days of age; (f, g): The relative abundance of *Proteobacteria* of ileum on 21 and 28 days of age respectively. JI7, JI21 and JI28 represent samples obtained from jejunum luminal contents of IUGR piglets on 7, 21 and 28 days of age; II21 and II28 represent samples obtained from ileum luminal contents of IUGR piglets on 21 and 28 days of age respectively. JN7, JN21 and JN28 represent samples obtained from jejunum luminal contents of NBW piglets on 7, 21 and 28 days of age; IN21 and IN28 represent samples obtained from ileum luminal contents of NBW piglets on 21 and 28 days of age respectively. Asterisks indicate different from corresponding NBW group: *P < 0·05; **P < 0·01.

To identify differences in microbial composition between the IUGR and NBW groups, Wilcoxon signed‐rank test was conducted. At the phylum level, the abundance of *Bacteroidetes* in the jejunum of the IUGR group was significantly lower than that in the jejunum of the NBW group (*P* < 0·05) at 7, 21 and 28 days of age (Fig. 5a–c), whereas the abundance of *Proteobacteria* was significantly higher (*P* < 0·05) in the ileum of the IUGR group than that in the ileum of the NBW group at 21 and 28 days of age (Fig. 5f,g). In addition, the relative abundances of *Firmicutes* in the ileum at 21 days of age and of *Spirochaeta* in the jejunum at 28 days of age were significantly lower (*P* < 0·05) in the IUGR group than the NBW group (Fig. 5d,e).

**Figure 6:**
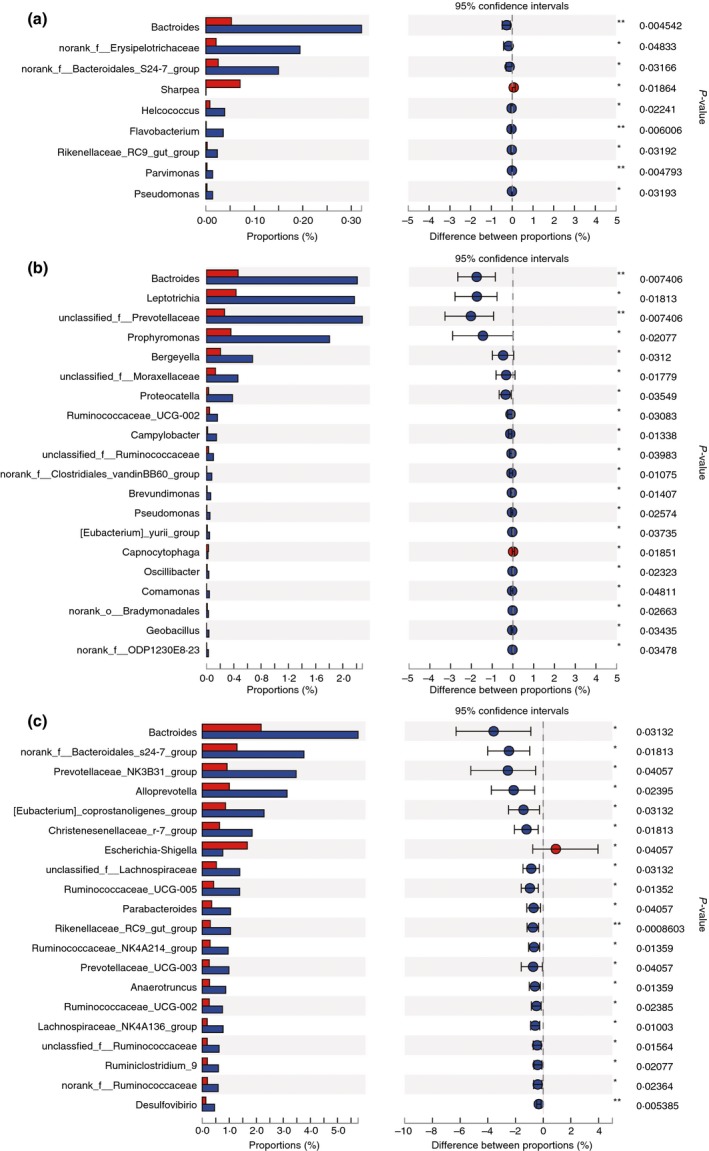
Differences in microbial abundance of jejunum between intrauterine growth retardation (IUGR) piglets and normal birth weight (NBW) piglets on 7 (a), 21 (b) and 28 (c) days of age at genus level. The top 20 abundance taxa are shown. Red represents IUGR piglets and blue represents NBW piglets. [Colour figure can be viewed at http://wileyonlinelibrary.com]

At the genus level, when compared to the NBW group, nine genera in the jejunum were less (*P* < 0·05) abundant in the IUGR group, including *Bacteroides*, no rank‐*Erysipelotrichaceae*, *Helcococcus*, *Flavobacterium* and *Parvimonas*; whereas the relative abundance of *Sharpea* in the IUGR group was higher (*P* < 0·05) at 7 days of age in the IUGR than the NBW group (Fig. 6a). The relative abundances *Bacteroides*, *Leptotrichia*, unclassified *Prevotellaceae*, *Porphyromonas* and *Bergeyella* were significantly lower (*P* < 0·05) in the IUGR group than the NBW group at 21 days of age (Fig. 6b). However, the abundance of *Escherichia–Shigella* in the IUGR group was significantly higher (*P* < 0·05), and that of 77 genera, such as *Prevotellaceae_NK3B31_group*, *Bacteroides*, *Alloprevotella*, *Prevotellaceae_UCG‐003* and *Ruminococcaceae_UCG‐005,* was significantly lower (*P* < 0·05) in the IUGR group than the NBW group at 28 days of age, (Fig. 6c).

**Figure 7:**
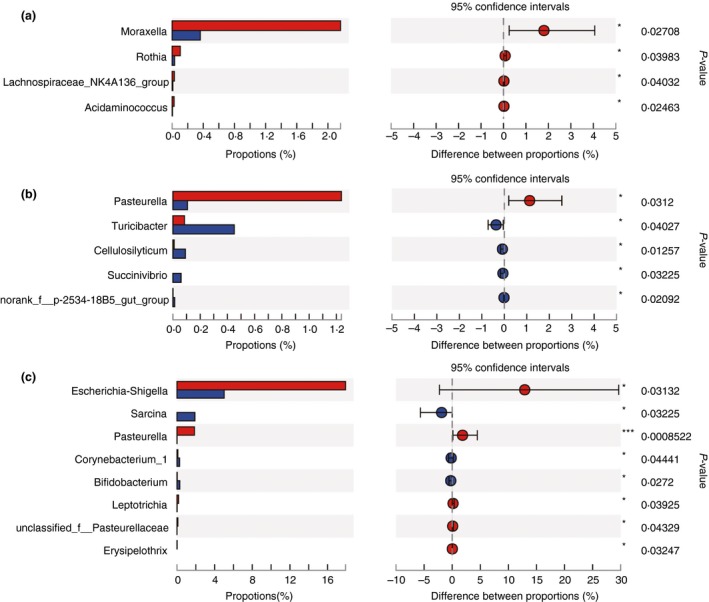
Differences in microbial abundance of ileum between intrauterine growth retardation (IUGR) piglets and normal birth weight (NBW) piglets on 7 (a), 21 (b) and 28 (c) days of age at genus level. The top 20 abundance taxa are shown. Red represents IUGR piglets and blue represents NBW piglets. [Colour figure can be viewed at http://wileyonlinelibrary.com]

The abundance of four genera was higher (*P* < 0·05) in the ileum of the IUGR group at 7 days of age than the NBW group, including *Moraxella*, *Rothia*, *Lachnospiraceae_NK4A136_group* and *Acidaminococcus* (Fig. 7a). Four genera had lower (*P* < 0·05) abundances: *Turicibacter*, *Cellulosilyticum*, *Succinivibrio* and no rank‐*p‐2534‐18B5_gut_group*, and *Pasteurella* had a higher (*P* < 0·05) abundance in the IUGR group than the NBW group at 21 days of age (Fig. 7b). Five genera had higher (*P* < 0·05) abundances: *Escherichia–Shigella*, *Pasteurella*, *Leptotrichia*, unclassified *Pasteurellaceae* and *Erysipelothrix*; and three genera: *Sarcina*, *Corynebacterium_1* and *Bifidobacterium* had lower (*P* < 0·05) abundances in the IUGR group than the NBW group at 28 days of age (Fig. 7c).

### Correlations between microbial biomarkers and body weight of newborn piglets

To understand the relationship between the intestinal microbiota and BW of newborn piglets, the spearman correlation between microbial relative abundance (based on the abundance of different taxa) and BW was analysed (Table 1). This showed that the abundance of 14 taxa was positively correlated with the BW, such as *Bacteroides*, *Bifidobacterium*, *Corynebacterium_1*, *Sharpea*, *Ruminococcaceae_UCG‐002*, *Turicibacter* and *Cellulosilyticum*, whereas the abundance of five taxa was negatively correlated with the BW: *Proteobacteria*, *Escherichia‐Shigella*, *Pasteurella*, *Leptotrichia* and *Erysipelothrix*.

**Table 1:** Correlations between microbial biomarkers and body weight of newborn piglets

Species	*ρ*	*P* values
*Oscillibacter*	0·76	0·001
*Ruminococcaceae_UCG‐002*	0·74	0·001
norank *Bradymonadales*	0·74	0·001
*Sharpea*	0·69	0·004
unclassified *Ruminococcaceae*	0·68	0·004
*Bacteroides*	0·64	0·011
*Bifidobacterium*	0·63	0·009
norank *ODP1230B8.23*	0·60	0·014
norank *Clostridiales_vadinBB60_group*	0·55	0·027
*Cellulosilyticum*	0·52	0·037
*Turicibacter*	0·52	0·041
*Corynebacterium_1*	0·51	0·042
unclassified *Family_XIII*	0·51	0·046
*Leptotrichia*	−0·52	0·040
*Proteobacteria*	−0·57	0·020
*Escherichia‐Shigella*	−0·57	0·020
*Erysipelothrix*	−0·57	0·021
*Pasteurella*	−0·76	0·001

### Profiles of microbial functions in the small intestine

**Figure 8:**
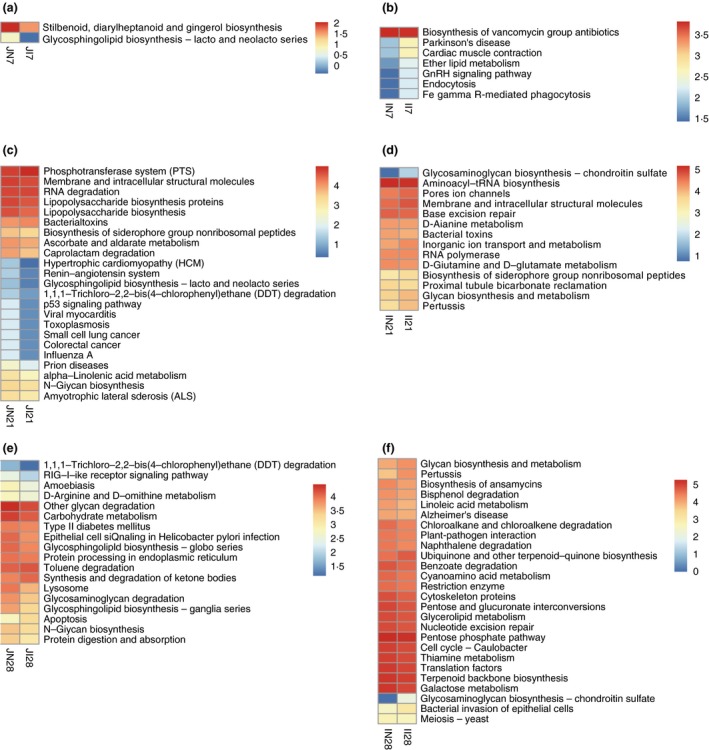
Difference in function of gut microbiota between intrauterine growth retardation (IUGR) piglets and normal birth weight (NBW) piglets. The average numbers of KEGG pathway (level 3) in each group were lg transformed and shown in the heatmap. (a), (c) and (e): Differences in abundance of jejunum microbial pathways between NBW and IUGR piglets on 7, 21 and 28 days of age respectively; (b), (d) and (f): Differences in abundance of ileum microbial pathways between NBW and IUGR piglets on 7, 21 and 28 days of age respectively. JI7, JI21, JI28, II7, II21 and II28 represent samples obtained from jejunum and ileum luminal contents of IUGR piglets on 7, 21 and 28 days of age respectively. JN7, JN21, JN28, IN7, IN21 and IN28 represent samples obtained from jejunum and ileum luminal contents of NBW piglets on 7, 21 and 28 days of age respectively. [Colour figure can be viewed at http://wileyonlinelibrary.com]

PICRUSt was used to estimate the functional capacity of the small intestine microbiota of newborn piglets. At 7 days of age, six pathways were enriched in the IUGR group compared to the NBW group: Parkinson’s disease, endocytosis, ether lipid metabolism, three downregulated pathways (stilbenoid, diarylheptanoid and gingerol biosynthesis), glycosphingolipid biosynthesis and biosynthesis of vancomycin group antibiotics (Fig. 8a,b). At 21 days of age, 10 pathways were enriched in the IUGR group compared to the NBW group, including the phosphotransferase system, bacterial toxins, RNA degradation, biosynthesis of siderophore group nonribosomal peptides and pores ion channels, and 27 pathways were downregulated, including lipopolysaccharide biosynthesis, biosynthesis of siderophore group nonribosomal peptides, alpha‐linolenic acid metabolism and N‐glycan biosynthesis (Fig. 8c,d). At 28 days of age, eight pathways were enriched in the IUGR group compared to the NBW group, including apoptosis, synthesis and degradation of ketone bodies, bacterial invasion of epithelial cells and pertussis, and 35 pathways were downregulated, including carbohydrate metabolism, protein digestion and absorption, galactose metabolism, glycerolipid metabolism and linoleic acid metabolism (Fig. 8e,f).

## Discussion

The trillions of intestinal microbes harboured in the mammalian gut profoundly influences the host’s health and disease status (Vaarala 2012; Frese *et al.* 2015; Lin *et al.* 2018). An increasing number of studies have shown that the small intestinal microbiota has a profound impact on host metabolism and growth (El Aidy *et al.* 2015; Burbach *et al.* 2017; Li *et al.* 2018). The present study characterized the small intestinal microbiota profiles of newborn piglets with IUGR. Our findings showed that the structure and function of the microbial community were highly changed in the IUGR piglets compared to the control subjects. In addition, the correlation analysis suggested that the abundance of 14 taxa was positively correlated with and that of five taxa was negatively correlated with the BW of newborn piglets.

Microbial diversity is highly associated with host health (Clarke *et al.* 2014; Batacan *et al.* 2017). A lower diversity of intestinal microbiota is considered a marker of dysbiosis in the gut (Duca *et al.* 2014) and contributes to a growing number of diseases, such as autoimmune diseases, obesity, inflammatory bowel disease and recurrent clostridium difficile‐associated diarrhoea (Ott *et al.* 2004; Chang *et al.* 2008). In the present study, the alpha diversity of the jejunum microbiota in IUGR piglets was significantly lower than that of control piglets at 7 and 21 days of age, which suggested that there is a dysbiosis in the gut of IUGR piglets. The IUGR piglets had lower relative abundances of *Bacteroidetes*, *Firmicutes* and *Spirochaeta*. *Bacteroidetes* are increasingly regarded as specialists for the degradation of high molecular weight organic matter, including proteins and carbohydrates (Thomas *et al.* 2011). Gut *Bacteroidetes* generally produce butyrate, which has antineoplastic properties and, thus, plays an important role in maintaining gut health (Kim and Milner 2007). Furthermore, *Bacteroidetes* also contributes to the host’s health, including by interacting with the immune system to activate T cell‐mediated responses (Mazmanian *et al.* 2008; Wen *et al.* 2008) and limiting the colonization of the gastrointestinal tract by potential pathogenic bacteria (Mazmanian 2008). Schwiertz *et al. *(2010) and Collado *et al. *(2008) demonstrated that overweight pregnant women and volunteers harboured significantly higher counts of the *Bacteroidetes* genus than normal‐weight pregnant women and lean volunteers. A recent study found that the relative abundance of *Firmicutes* is associated with energy intake from the diet (Turnbaugh *et al.* 2009); a higher proportion of the *Firmicutes* phylum was found in obese children and adults than lean individuals. In the present study, the lower relative abundance of *Bacteroidetes* and *Firmicutes* in IUGR piglets than controls suggests that IUGR piglets are less efficient at extracting energy from their diet and consequently gain less weight (Matheson *et al.* 2018). In addition, *Proteobacteria*, which includes a wide variety of pathogens (such as *Escherichia*, *Salmonella*, *Vibrio*, *Helicobacter* and *Yersinia*), is associated with inflammation (Cordonnier *et al.* 2016; Wang *et al.* 2019). In the present study, the IUGR piglets had a higher proportion of Proteobacteria, which increased continuously with age and negatively correlated with BW. These findings suggest that the IUGR piglets are more susceptible to disease.

At the genus level, we found more of a difference in the abundance of bacterial taxa in the jejunum than in the ileum between IUGR and NBW piglets. *Bacteroides* populations have the ability to harvest milk glycans and produce short‐chain fatty acids to stimulate the growth of intestinal epithelial cells, and thus, reduce the invasion and colonization by pathogens in nursing pigs (Marcobal *et al.* 2011; Marcobal and Sonnenburg 2012; Liu *et al.* 2018a). Previous studies have demonstrated a lower abundance of *Bacteroides* in obese than normal‐weight children (Sepp *et al.* 2013). In our study, *Bacteroides* was less abundant in the jejunum of IUGR than NBW piglets at 7, 21 and 28 days of age, and the abundance of *Bacteroides* positively correlated with BW. These differences may be due to our samples obtained from small intestinal contents instead of faeces. Therefore, the abundance of *Bacteroides* in the small intestine is important for the healthy growth of piglets. These findings are similar to a study by Zeng *et al. *(2015), which showed that low BW rex rabbits had a lower abundance of *Bacteroides* than controls. In addition, our study found that the proportions of several bacterial taxa belonging to the *Ruminococcaceae* family (e.g. *Ruminococcaceae_NK4A214_group*, *Ruminococcaceae_UCG‐004*, *Ruminococcaceae_UCG‐005*,* Ruminococcus_2* and *Oscillibacter*) were lower in the jejunum of IUGR than NBW piglets at 21 and 28 days of age. Previous studies have shown that the presence of *Ruminococcaceae* is related to the maintenance of gut health and the presence of numerous carbohydrate‐active enzymes that share a role as active plant degraders (Biddle *et al.* 2013). In addition, a higher abundance of *Ruminococcaceae* was detected in obese than normal weight mice (Kim *et al.* 2012). *Bifidobacteria* has been shown to be involved in nutrient metabolism and energy recycling, which play important roles in the trophic, metabolic and protective functions of the host (Blais *et al.* 2015; Azad *et al.* 2018). Our study showed that the abundance of *Oscillibacter* decreased in the jejunum of IUGR piglets at 21 days of age, as well as *Bifidobacterium* in the ileum of IUGR piglets at 28 days of age. In addition, the correlation analysis also revealed that *Oscillibacter* and *Bifidobacterium* were positively associated with BW. These findings suggest that IUGR piglets show low adaptability to the diet shift from solely sow milk to a solid diet, resulting in loss of BW. Furthermore, the abundance of *Escherichia–Shigella* and *Pasteurella*, opportunistic pathogens that causes a variety of infectious diseases in animals (Wilson and Ho 2013; Peng *et al.* 2016; Wang *et al.* 2019), was higher in IUGR than in NBW piglets. This suggested that these potential pathogens and opportunistic microbes might be related to the high susceptibility of IUGR piglets to disease. In order to explain the relationship between intestinal microbiota and host growth performance, it is necessary to further determine the intestinal health status.

Intrauterine growth retardation piglets had a lower abundance of microbial pathways related to carbohydrate metabolism, lipid metabolism, glycan biosynthesis and metabolism, amino acid metabolism, terpenoid and polyketide metabolism, xenobiotics biodegradation and metabolism, and biosynthesis of other secondary metabolites. The downregulated pathways of carbohydrate metabolism, lipid metabolism, and glycan biosynthesis and metabolism in IUGR piglets may be due to the presence of a lower abundance of *Bacteroidetes*, *Firmicutes* and *Bacteroides*, which can degrade high molecular weight organic matter (including proteins and carbohydrates), harvest milk glycans and produce SCFA (Marcobal *et al.* 2011; Thomas *et al.* 2011; Liu *et al.* 2018a). Stilbenoid, diarylheptanoid and gingerol, which are plant sources of phytoalexins, have natural anti‐inflammatory activities (Park *et al.* 1998; Yadav *et al.* 2003). The present study showed that IUGR piglets had several downregulated pathways related to xenobiotic biodegradation, including DDT, benzoate, bisphenol, chloroalkane, chloroalkene and naphthalene degradation. DDT is wildly contained in many pesticides, benzoate is used as a preservative in food and feed, and bisphenol is an endocrine‐disrupting chemical that exogenously interferes with the endocrine system of humans and animals; the endocrine imbalance of organisms exposed to bisphenol results in various abnormalities, such as genital disorders, abnormal behaviour, decreased reproductive capacity and larval death. The decreased abundance of xenobiotic biodegradation pathways in IUGR piglets suggests that exogenous substances cannot be effectively degraded, thereby damaging the host’s health.

## Author contributions

W.H.Z., P.F.X., X.D.W. and X.F.K. performed the experiments. W.H.Z., C.M., Q.Z., Y.L.Y. and X.F.K. wrote the manuscript. W.H.Z., Q.Z. and X.H.K. performed the statistical analyses. W.H.Z and P.F.X fed the animals. All authors reviewed the manuscript.

## Conflict of Interest

All authors have read and approved the final version of the manuscript and have declared that no competing interests exist.

## Supporting information


**Figure S1.** Difference of alpha diversity in the microbial communities between intrauterine growth retardation (IUGR) piglets and normal birth weight (NBW) piglets.

**Table S1.** Good's coverage for observed OTUs.
