# Supplementary figures and images for: Gut microbiota of newborn piglets with intrauterine growth restriction have lower diversity and different taxonomic abundances
Source: J Appl Microbiol. 2019 Jun 7;127(2):354–69. doi: 10.1111/jam.14304 (PMC6916403; doi:10.1111/jam.14304)

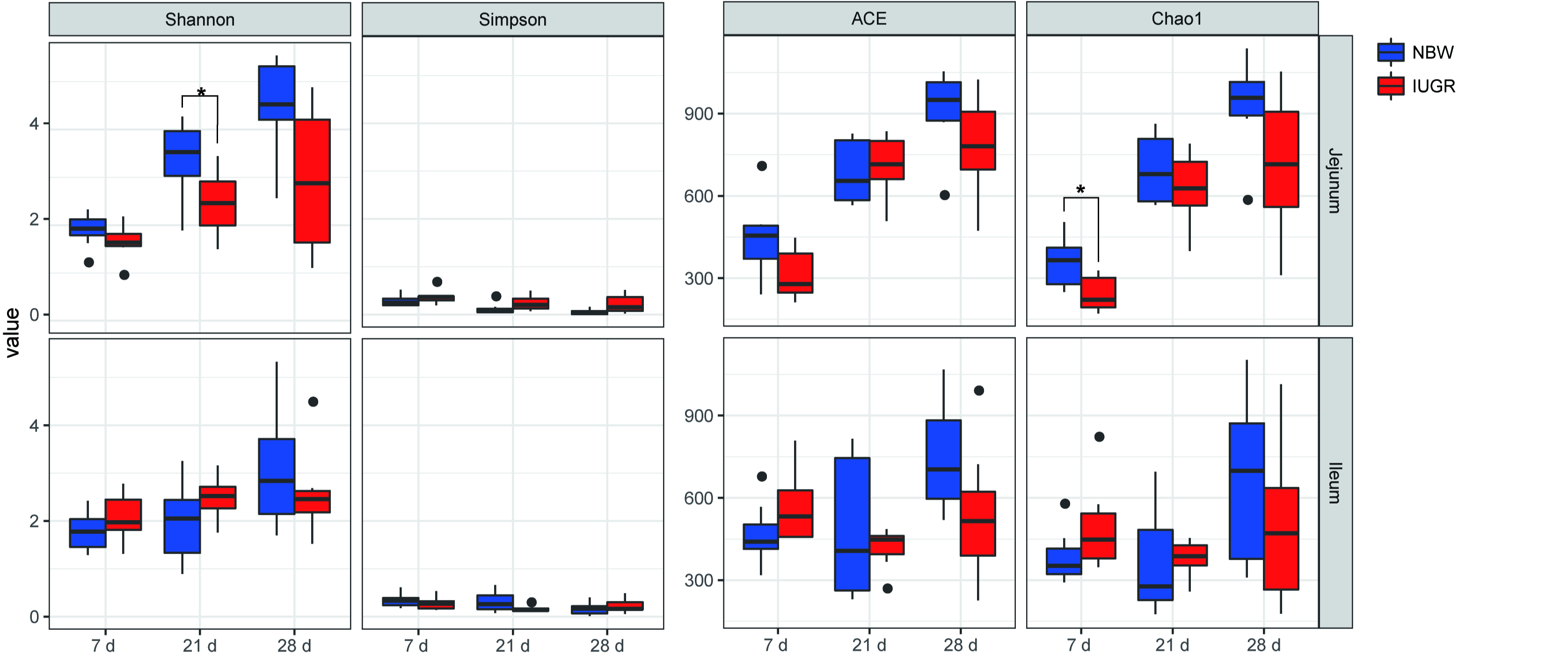

Supplement: Supplementary file 1 — Figure S1. Difference of alpha diversity in the microbial communities between intrauterine growth retardation (IUGR) piglets and normal birth weight (NBW) piglets. [file JAM-127-354-s001.tif]
