# Supplementary material for: Gut microbiota of newborn piglets with intrauterine growth restriction have lower diversity and different taxonomic abundances
Source: J Appl Microbiol. 2019 Jun 7;127(2):354–69. doi: 10.1111/jam.14304 (PMC6916403; doi:10.1111/jam.14304)
Supplement: Supplementary file 2 — Table S1. Good's coverage for observed OTUs. [file JAM-127-354-s002.docx]

**Supplementary Material**

# Supplementary Table

**Table S1 Good’s coverage for observed OTUs**

| Day of age | Jejunum | | Ileum | |
| --- | --- | --- | --- | --- |
|  | NBW | IUGR | NBW | IUGR |
| 7 | 99.701% ± 0.03% | 99.731% ± 0.06% | 99.691% ± 0.02% | 99.61% ± 0.03% |
| 21 | 99.48% ± 0.02% | 99.491% ± 0.03% | 99.71% ± 0.06% | 99.72% ± 0.02% |
| 28 | 99.411% ± 0.03% | 99.461% ± 0.04% | 99.51% ± 0.06% | 99.621% ± 0.07% |

IUGR: piglets with intrauterine growth retardation; NBW: piglets with normal birth weight.
